# Origin and Migration of Olfactory Cajal-Retzius Cells

**DOI:** 10.3389/fnana.2017.00097

**Published:** 2017-11-01

**Authors:** María Daniela Frade-Pérez, Amaya Miquelajáuregui, Alfredo Varela-Echavarría

**Affiliations:** Instituto de Neurobiología, Universidad Nacional Autónoma de México, Querétaro, Mexico

**Keywords:** development, telencephalon, embryo, LOT, reelin, mouse

## Abstract

Early telencephalic development involves the migration of diverse cell types that can be identified by specific molecular markers. Most prominent among them are Cajal-Retzius (CR) cells that emanate mainly from the cortical hem and to a lesser extent from rostrolateral, septal and caudo-medial regions. One additional territory proposed to give rise to CR cells that migrate dorsally into the neocortex lies at the ventral pallium, although contradictory results question this notion. With the use of a cell-permeable fluorescent tracer in cultured embryos, we identified novel migratory paths of putative CR cells and other populations that originate from the rostrolateral telencephalon at its olfactory region. Moreover, extensive labeling on the lateral telencephalon along its rostro-caudal extent failed to reveal a dorsally-migrating CR cell population from the ventral pallium at the stages analyzed. Hence, this work reveals a novel olfactory CR cell migration and supports the idea that the ventral pallium, where diverse types of neurons converge, does not actually generate CR cells.

## Introduction

Early corticogenesis is characterized by extensive cellular migration, both radial (from the ventricular zone (VZ) towards the pial surface) and tangential (along the pial surface; Marin and Rubenstein, 2001). Cajal-Retzius (CR) cells are one of the earliest neuronal types to be born at focal sites surrounding the cortical epithelium, from where they migrate tangentially to quickly populate the entire cortex. In spite of their transient existence (Chowdhury et al., 2010), CR neurons are crucial for lamination, arealization and aspects of connectivity in the cerebral cortex (Chao et al., 2009). Features that are traditionally used to identify these neurons include their typical tadpole cytomorphology, early birthdate, localization in the neocortical marginal zone, an excitatory (glutamatergic) profile, and expression of the markers Reelin and Calretinin (D’Arcangelo et al., 1995; Ogawa et al., 1995; Grkovic and Anderson, 1997; Meyer et al., 1999; Hevner et al., 2001, 2003a,b).

CR neuron subsets expressing different combinations of molecular markers, follow specific migration cues and routes that are associated with their generation sites at the septum, cortical hem, ventral caudo-medial telencephalic wall (vCMTW), choroid plexus and thalamic eminence (Meyer et al., 2004; Takiguchi-Hayashi et al., 2004; Bielle et al., 2005; García-Moreno et al., 2007, 2008; Tissir et al., 2009; Ceci et al., 2010, 2012; Miquelájauregui et al., 2010; Zimmer et al., 2010; Huilgol and Tole, 2016). Presumptive olfactory regions of the cortex, including the developing and early postnatal piriform and entorhinal cortices, contain subsets of neurons that are strikingly similar to the CR neurons found in the neocortex (Meyer et al., 2004; Yamazaki et al., 2004). These olfactory CR neurons may serve initially as guidepost neurons to direct lateral olfactory tract (LOT) formation, and then as reservoir of neocortical CR neurons during mid-embryonic development, as shown recently (Huilgol et al., 2013; Huilgol and Tole, 2016; de Frutos et al., 2016; Ruiz-Reig et al., 2017).

The olfactory system is special among other sensory systems in that information flows directly from the periphery without thalamic relays. Odor information is thought to be encoded in the piriform cortex, a trilaminar structure in the lateral cortex that receives sensory and associational projections from the olfactory bulb and intracortical regions, respectively (Suzuki and Bekkers, 2006, 2011; Diodato et al., 2016). Interestingly, inputs and outputs in the adult piriform cortex are cell-type specific and depend on the “molecular signature” of neurons, which is in turn specified early in embryonic development and possibly refined postnatally (Sarma et al., 2011; Suzuki and Bekkers, 2011; Pedraza and de Carlos, 2012; Carceller et al., 2016; Diodato et al., 2016). Although the developing piriform cortex has been relatively less studied, it has been shown that the inhibitory response to LOT stimulation and the activation of piriform cortex neurons do not emerge until the second postnatal week (Schwob et al., 1984; Meyer et al., 2006). Here we used genetic and cellular labeling methods to characterize the origin and migration of subsets of olfactory CR neurons in the rostral and lateral telencephalon. First, we identified two novel streams of olfactory neurons originating in the rostrolateral telencephalon close to the prospective olfactory bulbs. One of them, labeled specifically at E11, migrated dorsally towards the dorsal pallium. The other stream, which migrated ventro-caudally towards the piriform cortex, was present in *Lhx5* knock-out embryos and, unlike septal Er81^+^ CR neurons, was unaffected by the inhibition of FGFs (Zimmer et al., 2010). We also confirmed earlier findings that Reelin-expressing cells originating from the ventral pallium/pallial-subpallial boundary (VP/PSB) converge in the prospective piriform cortex (Ceci et al., 2012). We thus confirmed previous studies and added novel information about the complex patterns of olfactory CR neuron development.

## Materials and Methods

### Animals

CD-1 wild type mice and a transgenic line carrying an *Lhx5* null allele bred into the CD-1 background (Zhao et al., 1999) were used. This study was carried out with the approval of the Research Ethics Committee of the Instituto de Neurobiología, UNAM (Protocol #001) and according to the technical specifications for production, care, and use of laboratory animals of the Mexican government (NOM-062-ZOO-1999). The day of detection of vaginal plug was considered as embryonic day 0.5 (E0.5).

### Whole Embryo Culture

Pregnant dams were anesthetized with a Ketamine-Xylazine mixture (80 and 30 mg/Kg respectively); individual E10.5–12 embryos and their attached placentas were carefully extracted and cultured in toto, as described by de Carlos et al. (1996). Cell migration was assessed by injection of the cell-permeable dye carboxy-fluorescein diacetate succinimidyl ester (CFDA-SE, V12883 Invitrogene, Waltham, MA, USA) at the ventricular lining of the right telencephalic vesicle using an air-driven pulse injector through a glass pipette. Injected embryos were cultured for 24 h in glass bottles (2–3 embryos/bottle) containing 4 ml of pre-warmed and oxygenated rat or fetal bovine serum (16000044, Gibco, Grand Island, New York, NY, USA) supplemented with 2 mg/ml glucose and 1% of a mixture of penicillin-streptomycin (15070–063, Gibco). To prepare rat serum, whole blood was collected from the inferior cava vein, placed in 15 ml polypropylene tubes on ice until clot formation, followed by clot removal, centrifugation (5000× *g* for 15 min), serum collection (using a Pasteur pipette), complement inactivation (1 h at 56°C) and storage at −70°C until use.

For whole embryo cultures, individual bottles were inserted in a custom-made rotator device placed within an incubator (35°C) with constant individual flow of a gas mixture composed of 95% O_2_ and 5% CO_2_. Serum was replaced every 12 h. For FGF inhibition experiments, serum was supplemented with 10 μM of SU5402 (572630, Calbiochem, Billerica, MA, USA) dissolved in DMSO (9224–01 J.T.Baker Center Valley, PA, USA) with an equivalent volume of DMSO used in control cultures.

### Tissue Preparation

Pregnant dams were anesthetized and killed by cervical dislocation. Embryos collected or previously cultured were dissected in cold PBS and fixed for 16 h in 4% paraformaldehyde (PFA) in PBS at 4°C. Embryos were then washed with PBS, their brains isolated and cryoprotected in 30% sucrose in PBS for at least 16 h at 4°C, followed by immersion and freezing in Tissue-Tek O.C.T. (Sakura Finetec 25608–930, VWR, Radnor, PA, USA). Cryostat sections (15–20 μm thick) were collected on Superfrost Plus slides (8311703 VWR), air-dried for 2 h and stored at −20°C until use. The results shown are from at least three embryos per stage and per condition (culture, CFDA labeling, immunostaining or *in situ* Hybridization (ISH); Wild-Type (WT) or mutant).

### Immunohistochemistry (IHC)

Cryostat sections (20 μm) were washed with PBS, blocked for 1 h with 5% goat serum (16210072, Gibco, New Zealand origin) in PBS and then incubated in the following primary antibodies: anti-Reelin (1:3000, MAB5364, Millipore, Billerica, MA, USA), anti-Tbr1 (1:1000, AB31940, ABCAM, Cambridge, MA, USA) or anti-Calbindin (1:1000, AB1778, Chemicon, Temecula, CA, USA) for 16 h at 4°C in PBS containing 5% goat serum and 0.1% Triton X-100. Sections were subsequently washed with PBS and a second blocking step was performed. Secondary antibody incubation (1:1000) was performed with Anti-mouse-Cy3 antibodies (115–166–003, Jackson Immunoresearch, Bar Harbor, ME, USA), Anti-mouse Cy5 antibodies (115–175–146, Jackson Immunoresearch) or Anti-rabbit Cy3 antibodies (111–166–003, Jackson Immunoresearch) in PBS containing 5% goat serum and 0.1% Triton X-100 for 1 h at room temperature. Sections were then washed with PBS and mounted in Mowiol mounting medium [9% Mowiol 4–88 (475904 Calbiochem, Billerica, MA, USA), 25% Glycerol, 100 mM Tris pH 8.5].

### *In Situ* Hybridization (ISH)

Digoxygenin-labeled riboprobes were synthesized by *in vitro* transcription from plasmids in which we cloned: *Er81* cDNA sequence corresponding to a 900 bp fragment and *Dbx1* cDNA corresponding to a 890 pb fragment located in the fourth exon.

Whole brain ISH was performed as described in Varela-Echavarría et al. (1996). Briefly, brains were treated for 5 min in each of a series of methanol solutions in PBS (25%, 50%, and 75%), 5 min in 100% methanol and later in the same series in decreasing concentrations, and clarified by immersing in 6% hydrogen peroxide in PBS for 15 min. Brains were then treated with proteinase K (25530015 Invitrogene, Waltham, MA, USA) and fixed with 4% PFA in PBS. Embryos were washed with PBS which was then substituted with hybridization buffer (50% Formamide, 5× SSC pH 4.5, 50 μg/ml Heparin, 0.1% Tween, 50 μg/ml Yeast RNA, 50 μg/ml Salmon sperm) and 1 μg/ml denatured riboprobe was then added, followed by incubation at 70°C for 14–18 h. Unbound probe was washed 3 times for 1 h each in hybridization buffer. Tissue was then incubated at room temperature with anti-digoxygenin Fab antibody fragments coupled to alkaline phosphatase (11093274910 Roche, Basel, Switzerland) and developed in BM purple solution (11442074001 Roche) in the dark until color emerged.

### Image Acquisition and Analysis

Whole brain bright-field and fluorescence images were obtained using a Nikon Eclipse E-600 microscope. Confocal images (1 μm) were obtained using a Carl Zeiss LSM510 META confocal microscope. ISH images were obtained with a Carl Zeiss STEMI 2000-C microscope using an Optronics camera. Co-localization analyses were performed on confocal micrographs with the open-source software ImageJ^1^ by counting cells expressing the various molecular markers among CFDA-labeled cells that could be distinguished away from the labeling site and located in the marginal zone of 20 μm coronal brain slices of each embryo analyzed. To do this, a confocal image was obtained from the middle region along the Z-axis of each section of a series containing CFDA-labeled cells. Only cells of average or near-average size were counted.

## Results

### The Rostrolateral Pallium Gives Rise to Two Streams of Cells that Migrate Towards the Dorsal Cortex and the Piriform Cortex

**Figure 1:**
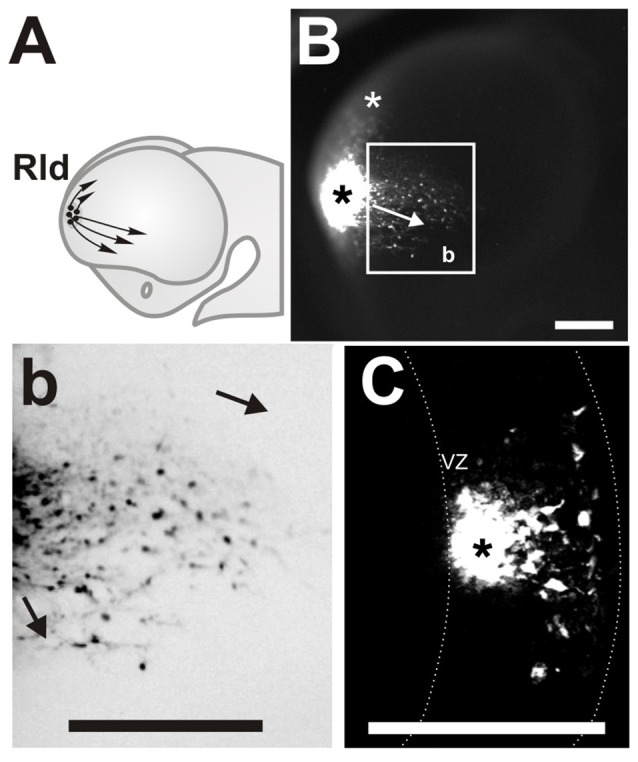
Rostro Lateral Domain (Rld): a rostral site of cell generation with different migration routes. **(A)** Scheme of a developing forebrain showing the cells derived from the (Rld, black dots) and the dorsal and ventral directions of migration (arrows). **(B)** Lateral view of a telencephalic vesicle (E11.0 + 24 h in culture) showing a site of carboxy-fluorescein diacetate (CFDA) labeling at the Rld (black asterisk), labeled cells migrating in caudal and ventral directions (arrow in inset) and cells displaced dorsally (white asterisk). **(b)** Inverted color magnification of inset in **(B)** showing cells migrating in caudal and ventral directions. **(C)** Representative telencephalic coronal section showing the labeled site in the VZ (asterisk) and cells migrating radially towards the pial surface. Dotted line in **(C)** limits the borders of the neocortex in coronal sections. VZ: ventricular zone. Scale bar: 200 μm.

To study tangential cell migration routes in the developing mouse telencephalon, we labeled several rostral and lateral proliferative regions (i.e., at the VZ) with CFDA, a cell-permeable tracer which fluoresces upon cell entry. The labels were made from the vicinity of the prospective olfactory bulbs to the most caudal areas of the telencephalic vesicles in E10.5 to E11.5 embryos. After labeling, embryos were cultured into for 24 h revealing both known and novel migrating populations (Figure 1).

Labeled sites at a rostral lateral domain (Rld) adjacent to the prospective OB territory (Figure 1A, black dots) generated cells that migrated in two main directions (Figure 1A, arrows). Approximately 20% of all labeled cells migrated away from the injection site (Figure 1B, black asterisk) in a dorsal direction reaching the dorsal pallium after 24 h (Figure 1B, white asterisk). We consistently identified this migrating population only in injections made at E11. Most migrating cells from this region followed caudal and ventral directions oriented toward the piriform cortex and olfactory tubercle, respectively (Figures 1B,b, arrows). This caudoventral migration pattern was observed in all labeled embryos of stages E10.5 to E11.5. We were also able to confirm that CFDA was specifically applied in the VZ (Figure 1C, asterisk) of the Rld, from where labeled cells migrated radially to the pial side of the telencephalic vesicle followed by tangential displacement. Thus, these results reveal novel migratory cells originating in the rostral lateral region of the developing telencephalon.

### The Rld Generates Cajal-Retzius Neurons and Other Migratory Cell Groups

**Figure 2:**
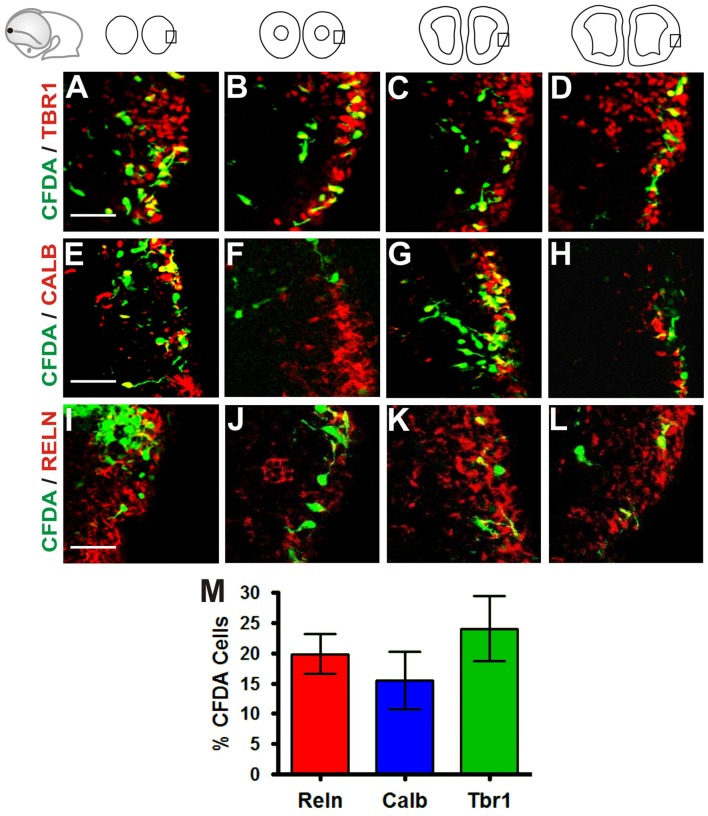
Neurons generated in the Rld correspond to various cell populations. Immunostaining of telencephalic coronal sections from a representative CFDA-labeled embryo (E10.5 + 24 h in culture) with antibodies against different markers. **(A–D)** Pallial marker Tbr1. **(E–H)** Subpallial marker Calbindin. **(I–L)** Reelin, marker of Cajal-Retzius (CR) cells. **(M)** Proportions of CFDA-labeled neurons positioned in the MZ near to the piriform cortex: approximately 24% are Tbr1^+^, 15.5% are Calbindin^+^ and 19.8% are Reelin^+^. Scale bar: 50 μm.

**Figure 3:**
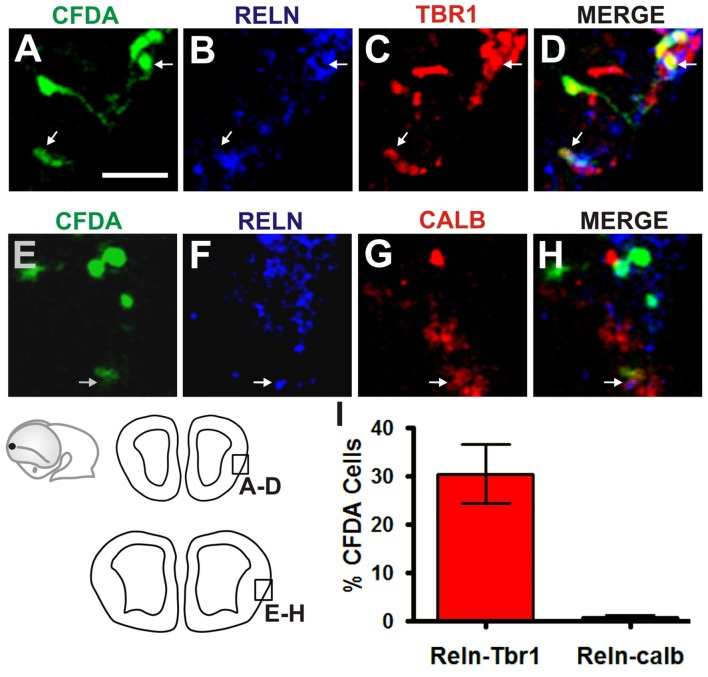
Olfactory CR neurons derived from the Rld. Double immunostaining of telencephalic coronal sections from a representative CFDA-labeled embryo (E10.5 + 24 h in culture). Shown are labelings with CFDA (green) and antibodies against Reelin (blue) and Tbr1 or Calbindin (red) in rostral **(A–D)** and caudal **(E–H)** sections. **(I)** Cell quantitation in the MZ of Reelin and Tbr1 co-labeled neurons (30.4%). Fewer than 0.7% expressed both Reelin and Calbindin. Scale bar 50 μm.

To characterize further the identity of the cell populations generated at the Rld, we performed IHC on coronal sections of telencephalic vesicles of embryos labeled with CFDA at E10.5 with antibodies that stain specific neuronal populations. We used antibodies for Tbr1 (Figures 2A–D) which is expressed by cells of pallial origin from early developmental stages; Calbindin (Figures 2E–H), a marker of neurons of subpallial origin; and Reelin (Figures 2I–L), expressed in the telencephalic marginal zone by CR cells (Bulfone et al., 1995; Grkovic and Anderson, 1997; Meyer et al., 1999; Hevner et al., 2001). We observed that most of the CFDA-labeled cells that expressed these markers were located in the marginal zone. Approximately 24% of CFDA-labeled cells were also labeled by Tbr1, 15.5% by Calbindin and 19.8% by Reelin (Figure 2M). As CR cells were also expected to co-express both Reelin and Tbr1, we performed double immunostaining of CFDA labeled cells and found that 30.4% of Reelin-positive neurons co-expressed Tbr1 (Figures 3A–D,I), whereas less than 0.7% co-expressed Calbindin (Figures 3E–I). Our results thus indicate that cells generated at E10.5 from the Rld represent different cellular subsets including Reelin-expressing cells, about a third of which seems to correspond to CR cells.

### Rld-Derived Reelin^+^ Neurons Constitute a Novel Migratory Population

We assessed whether the Reelin-expressing cell population generated at the Rld corresponds to a previously described cell group. Previous reports have shown that subsets of Reelin-expressing neurons originate at the vicinity of the septum in the medial telencephalic wall (Bielle et al., 2005; García-Moreno et al., 2008; Ceci et al., 2012) and in the rostromedial pallium (Zimmer et al., 2010). CR neurons from the latter express the transcription factor Er81 and their generation depends on FGF8 signaling (Zimmer et al., 2010).

**Figure 4:**
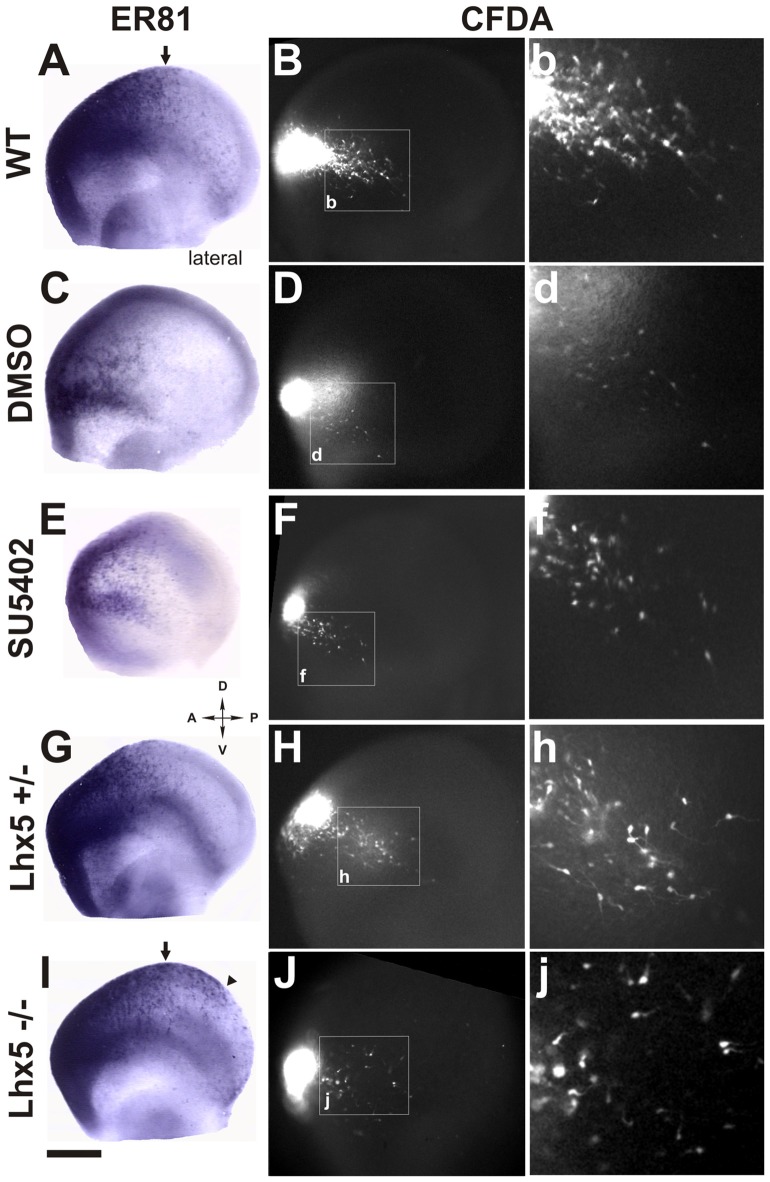
Rld-derived cells do not belong to *Er81* or *Lhx5* CR cell populations. *Er81 in situ* hybridization (ISH) was performed in CFDA-labeled embryos (E11.5 + 24 h in culture) in several conditions. **(A)** Wild-type (WT) embryo showing *Er81* expression in rostrolateral and rostrodorsal areas. **(B,b)** Migration of CFDA-labeled cells in a caudo-ventral direction. **(C,D,d)** Control embryo (cultured in 0.1% DMSO) showing similar pattern of Er81 expression and direction of migration of CFDA labeled cells as in **(A,B)**. **(E,F)** Treatment with the FGF inhibitor SU5402 showing decreased *Er81* expression **(E)** and a reduction in the caudoventral migration of CFDA^+^ migrating cells **(F,f)**. **(G,H,h)** Heterozygous *Lhx5* mouse embryo showing patterns of both *Er81* expression and CFDA-labeled cell migration similar to WT controls **(A,B)**. **(I)** *Lhx5* knock-out mouse embryo showing an expansion of *Er81* expression in dorsocaudal regions. **(J,j)** *Lhx5* mutants showing a slight decrease in the amount of CFDA-labeled cells, and although migration in the caudoventral direction was present, cells appeared to be disorganized. Arrows in **(A,I)** show the normal limit of *Er81* expression; arrowhead in **(I)** shows expanded *Er81* expression in *Lhx5* mutants **(I)** and asterisk shows some dispersed points of *Er81* expression. Scale bar: 200 μm.

To characterize the cells generated at the Rld, we performed* ISH* for *Er81* in E11.5 telencephalic vesicles. As expected, we observed strong *Er81* expression in the dorsorostral cortex, including the Rld domain, as well as in the ventral pallium (Figure 4A). Interestingly, we observed that subsets of CFDA-labeled cells originating in the Rld migrated across *Er81*^+^ regions (Figures 4B,b). To determine whether the CFDA^+^ cells originating in the Rld belong to the Er81^+^ population described before (Zimmer et al., 2010), we performed *Er81* ISH in E10.5 embryos labeled with CFDA-and cultured them for 24 h in the presence of the FGF inhibitor SU5402. Since Er81^+^ CR neuron specification requires FGF signaling and SU5402 affects the migration of these neurons in cortical explants, we hypothesized that the migration of neurons from the Rld would be at least partially impaired. First, we observed a dramatic reduction in *Er81* expression upon pharmacologic inhibition of FGF (Figure 4E) that was not evident in control cultures (Figure 4C). However, the migration of CFDA-labeled cells from the Rld did not seem to be impaired by FGF inhibition, when compared to DMSO controls (Figures 4D,d,F,f). These results suggest that the CFDA-labeled cells generated from the Rld (at least at E10.5 + 24 h), do not belong to the Er81^+^ subset of CR neurons (Zimmer et al., 2010). Instead, we postulate that these Reelin-expressing cells are a population that has yet to be characterized.

### Migration of Rld-Derived Cajal-Retzius Neurons Is Unaffected in *Lhx5* Mutants

Our group has previously reported that the *Lhx5* homeodomain transcription factor is required for the normal development of CR neurons and that its absence leads to decreased Reelin expression in embryonic sites of CR generation such as the cortical hem, septal and VP regions, as well as in the dorsal pallium (Miquelájauregui et al., 2010). In order to investigate whether the generation or migration of rostral CR neurons from the Rld depend on *Lhx5* function, we analyzed *Er81* expression in *Lhx5* knock-out embryos at E11.5. As shown in Figure 4, *Er81* expression was detected in the expected rostral locations in both *Lhx5* mutants and controls. However, we noticed that the Er81^+^ dorsal domain extends caudally in *Lhx5* mutants beyond the normal distribution observed in WT or heterozygous embryos (Figures 4A,G,I). To determine whether the migration of cells originating in the Rld is affected by the lack of *Lhx5*, we performed rostral CFDA-labeling and embryonic culture of heterozygous and Lhx5 knock-out. Cells originating in the Rld of WT control (Figures 4B,b) and heterozygous (Figures 4H,h) embryos showed comparable migration patterns while cells of mutant embryos were more dispersed (Figures 4J,j). Although the extent of the caudal migration from the Rld did not seem to be affected, CFDA-labeled cells seemed less confined to the PSB (Figures 4H,h,J,j). These results suggest that Rld-derived CR olfactory neurons are able to migrate in the absence of Lhx5, but dispersed out of limits of the normal migratory stream.

### The Ventral Pallium (VP) Gives Rise to Ventrally–but Not Dorsally-Migrating Cells

We analyzed a lateral region in the ventral pallium at the pallial-subpallial boundary (VP/PSB) where diverse cell types are generated, based on the expression of cell identity markers (Bielle et al., 2005; García-Moreno et al., 2008; Ceci et al., 2012). Neocortical CR neurons are amongst the populations proposed to be generated in the VP/PSB, as assessed by genetic labeling of Dbx1^+^ lineage progenitors and cell lineage tracing in slice cultures (Bielle et al., 2005). A recent study using whole-embryo cultures, however, failed to detect such dorsal migration when lineage tracers were applied to this location (Ceci et al., 2012).

**Figure 5:**
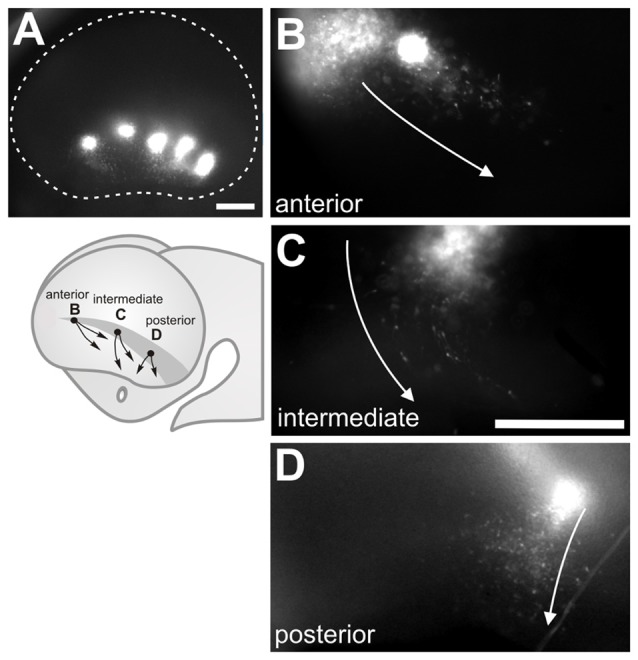
Cells generated at the ventral pallium (VP) migrate ventrally. **(A)** Multiple CFDA-labeling along the antero-posterior axis of the VP in E11.5 embryos showing a ventral direction of migration. **(B–D)** Single CFDA labelings (in different embryos) at different positions (arrows depict direction of migration): **(B)** Anterior VP, **(C)** Medial VP, **(D)** Posterior VP. Scale bar: 200 μm.

For a thorough analysis regarding this controversial region, we performed extensive CFDA labeling along its rostral-caudal axis in single or multiple simultaneous labels in mouse embryos from E10.5 to E12.0 and cultured them in toto for 24 h (Figure 5A and data not shown for E12.0). Most cells generated in the rostral VP migrated in a caudo-ventral direction (Figure 5B) while cells generated in the intermediate VP had a tendency to migrate either ventrally or caudally (Figure 5C). Finally, cells labeled in the posterior VP migrated predominantly into the ventral telencephalon (Figure 5D). In addition, we observed that when CFDA was injected just dorsal to the VP, most labeled cells migrated ventrally into the VP without crossing it, at least after 24 h of culture. When CFDA was applied ventral to the VP, labeled cells followed ventral routes (data not shown). In addition, labeling in the lateral ganglionic eminence in the ventral pallium revealed cells that migrate to fill the piriform cortex and olfactory tubercle (García-Moreno et al., 2008; Ceci et al., 2012). Therefore, our results are consistent with previous observations made by García-Moreno et al. (2008) as well as Ceci et al. (2012) who described that cells generated by the germinative zone of the dorsolateral telencephalon tend to migrate into the olfactory cortex and that cells originating in the VP fail to follow dorsal migratory routes as proposed by Bielle et al. (2005), at the stages examined.

**Figure 6:**
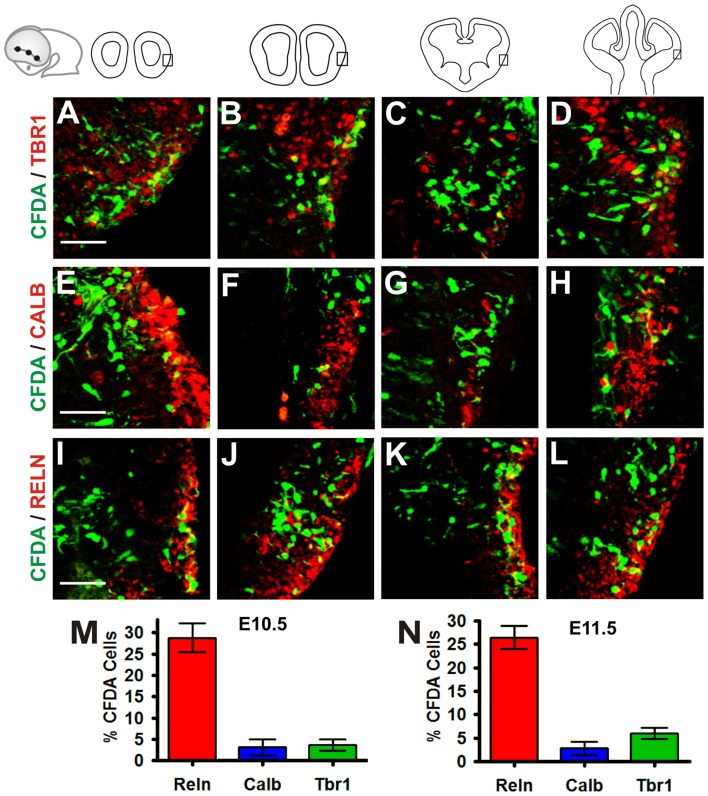
Cells generated in the VP belong to different subpopulations of both pallial and subpallial origin. Immunohistochemistry (IHC) of telencephalic coronal sections from a representative cultured embryo (E11.5 + 24 h in culture) with antibodies to different markers: **(A–D)** pallial marker Tbr1, **(E–H)** subpallial marker Calbindin and **(I–L)** Reelin, marker of CR cells. **(M,N)** Proportion of CFDA^+^ cells at E10.5 + 24 h in culture **(M)** and E11.5 + 24 h in culture **(N)** stages. At E10.5 + 24 h in culture, approximately 3.6% of CFDA-labeled cells expressed Tbr1, 3.1% expressed Calbindin, and 28.7% expressed Reelin **(M)** In E11.5 + 24 h in culture, 6% of labeled cells expressed Tbr1, 2.8% expressed Calbindin and 26.4% expressed Reelin **(N)**. Scale bar: 50 μm.

To characterize further the migratory cells generated in the VP, we performed IHC using antibodies for Tbr1, a putative pallial marker (Figures 6A–D), Calbindin, a putative subpallial marker (Figures 6E–H) and Reelin, a marker of CR neurons at embryonic stages (Figures 6I–L) in E10.5 and E11.5 embryos cultured for 24 h (Bulfone et al., 1995; Grkovic and Anderson, 1997; Meyer et al., 1999; Hevner et al., 2001). Our analysis revealed that in E10.5 embryos, approximately 3.6% of CFDA cells labeled at the VP expressed Tbr1, 3.1% expressed Calbindin, and 28.7% expressed Reelin (Figure 6M). In E11.5 embryos in culture 6% of labeled cells expressed Tbr1, 2.8% expressed Calbindin, and 26.4% expressed Reelin (Figure 6N). These results indicate that cells arising from the VP at E10.5 and E11.5 belong to different subpopulations of both pallial as subpalial origin. In agreement with Ceci et al. (2012), a third of these cells were Reelin-positive, suggesting that this cell population is the same analyzed by these authors.

### Novel Sites of *Dbx1* Expression that Could Give Rise to Cajal-Retzius Cells in the Developing Telencephalon

The contradictory findings of Bielle et al. (2005) with those of Ceci et al. (2010) and our own initial findings, prompted us to analyze this issue further. The CR population proposed to be generated from the VP/PSB region is putatively derived from Dbx1-expressing progenitor cells described to be present in this region, the fate of which was followed in mice by *Dbx1*-dependent genetic labeling (Bielle et al., 2005). Since our results and those of Ceci et al. (2012) did not reveal Reelin-expressing cells that migrated dorsally from this region, we hypothesized that the CR cells derived from *Dbx1* progenitors could be generated in *Dbx1* expression domains located elsewhere in the telencephalon.

**Figure 7:**
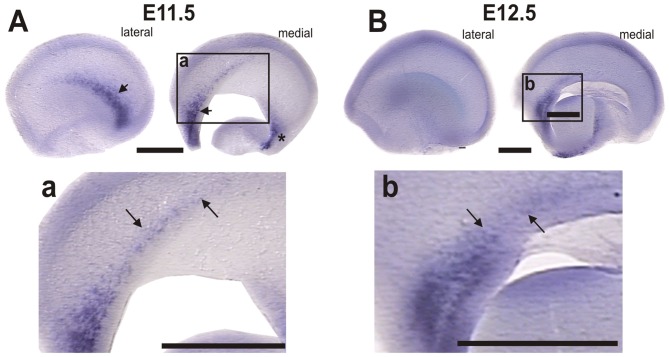
*Dbx1* is expressed in the rostral-most region of the cortical hem. *Dbx1 ISH* in WT mouse telencephalic vesicles. **(A)** E11.5 lateral and medial views; arrows indicate *Dbx1* expression in the olfactory region and septum; asterisk indicates the caudomedial side. **(a)** Higher magnification of inset in **(A)**; arrows indicate *Dbx1* expression in the rostral end of the prospective cortical hem. **(B)** E12.5 lateral and medial side of telencephalic vesicles, showing reduced *Dbx1* expression. **(b)** *Dbx1* expression persists in the septum and in the rostral end of the cortical hem. Scale bar: 200 μm.

To address this possibility we analyzed *Dbx1* expression by ISH (Figure 7). At E10.5 *Dbx1* expression was limited to small lateral and septal expression domains (data not shown). At E11.5, we detected expression in the septum and the lateral territory of the olfactory region (Figure 7A, arrow) as described by Bielle et al. (2005). Additional domains, however, were detected in the dorsal septal region at the rostral end of the prospective cortical hem (Figure 7A, arrows) and in a ventral domain in the caudo-medial telencephalic wall (Figure 7A, asterisk). Moreover, in E12.5 embryos we observed that *Dbx1* expression was greatly reduced in the lateral side of the telecephalon (Figure 7B) while it remained in the septum and rostral end of the cortical hem (Figure 7b, arrows).

With the exception of the olfactory region, all these regions containing *Dbx1* expressing cells have been shown to give rise to Reelin-expressing cells that migrate via dorsal and ventral routes to occupy lateral telencephalic regions including the neocortex (Soriano and Del Río, 2005; García-Moreno et al., 2008; Miquelájauregui et al., 2010; Ceci et al., 2012). This is especially relevant for the prospective cortical hem which gives rise to most CR cells. Hence, it is possible that these medial regions expressing *Dbx1* give rise to CR cells.

## Discussion

In this work we analyzed cell populations generated in the VZ of lateral regions of the developing telencephalon. Two areas were the focus of this work. The first was a rostral domain in the proximity of the territory that gives rise to the olfactory bulbs, which we refer to as the Rld. The second was an elongated domain in the olfactory region adjacent to the pallium/subpallium boundary, the VP. From these regions, cells migrate radially toward the pial surface, and then tangentially towards their final destination. We detected a population of cells originating in the Rld that migrates dorsally and one that migrates in a caudo-ventral route that have not been characterized thus far. On the other hand, we observed cells originating from the VP that migrate predominantly in a ventral direction and appear to correspond to a population described previously (García-Moreno et al., 2008; Ceci et al., 2012). Moreover, systematic attempts to detect cells that migrated dorsally from this region as proposed by Bielle et al. (2005), were unsuccessful at the stages examined.

### Rld: An Origin Site of Various Cell Populations

Diverse migratory populations have been identified emanating from various rostral telencephalic regions. Labeling with fluorescent lineage tracers has revealed sites of CR cell origin such as the rostromedial telencephalic wall (García-Moreno et al., 2008), the septum (Bielle et al., 2005; Ceci et al., 2012) and the septoeminential sulcus (García-Moreno et al., 2008). The caudo-ventrally migrating population from the Rld described in this work that represents approximately 80% of all labeled cells corresponds to a novel population, one fifth of which expresses Reelin. Expression of this marker and their location in superficial regions of the developing cortex suggest that these cells are part of the CR repertoire. It is noteworthy that a rostrolateral population of *Er81*-expressing cells dependent upon FGF signaling comprise CR cells that occupy the dorsal cortex (Zimmer et al., 2010). Since the Rld-derived cell group identified herein is not affected by a treatment that impairs FGF signaling, we propose that these cells belong to different populations. Furthermore, the absence of *Lhx5*, which causes an overall reduction in CR cell abundance and aberrant migration of Reelin-expressing cells (Miquelájauregui et al., 2010), affects dramatically the distribution of *Er81*-expressing cells without an apparent effect on the Rld population. Interestingly, the dorsally-migrating population was detected only when the labeling was done in E11.0 embryos, revealing an ontogenetic time-window for their generation.

The caudoventral migration of cells from the Rld into the olfactory region we observed is consistent with previous findings that revealed this last region as a site of convergence of various migratory populations (Bielle et al., 2005; García-Moreno et al., 2008; Zimmer et al., 2010; Ceci et al., 2012). For example, cells originating in the VP/PSB migrate ventrally reaching the prospective piriform cortex (Bielle et al., 2005). Moreover, cells from the dorsal telencephalon, lateral ganglionic eminence, septoeminential sulcus, rostral medial telencephalic wall (García-Moreno et al., 2008), ventral pallium and septum (Ceci et al., 2012) follow various routes to reach the piriform cortex and olfactory tubercle (García-Moreno et al., 2008; Ceci et al., 2012). About a third of Reelin-expressing cells migrating from the Rld were found to express Tbr1 while a small fraction expressed Calbindin. This is consistent with previous observations showing that both piriform cortex and olfactory tubercle receive excitatory neurons from the septum and more dorsal regions (García-Moreno et al., 2008; Ceci et al., 2012; Huilgol and Tole, 2016). Interestingly, the convergence of migratory Reelin-expressing neurons in the piriform cortex has been observed in the opposite direction i.e., Reelin^+^ cells born in the caudomedial telencephalic wall and migrating via a ventral path around the caudal pole of the telencephalon towards the prospective olfactory cortex Takiguchi-Hayashi et al., 2004; Miquelájauregui et al., 2010; Huilgol et al., 2013). Hence, the Rld gives rise to a migratory stream that seems to be independent of FGF signaling and Lhx5 function.

### Cells Generated in the VP Do Not Migrate Dorsally

In addition of representing a convergence site for migratory populations, the lateral aspect of the olfactory region is itself a source of migratory cells. Based on genetic labeling of Dbx1^+^ neural progenitors and fluorescent tracing in cultured slices from embryonic telencephalon, Bielle et al. (2005) proposed that subsets of CR cells originate in the VZ of the VP/PSB and the septum, from where they migrate dorsally to the neocortex. Through genetic ablation of *Dbx1* they also observed a dramatic reduction of CR cells. A different study using florescent tracers in whole-embryo cultures, however, revealed that VP-derived cells migrate ventrally, but not dorsally, out of this region (Ceci et al., 2012). To address these contradictory results, we performed VP labeling with fluorescent tracers in E10.5 to E12.0 embryos followed by culture for 24 h. Extensive labeling along the whole antero-posterior extent of the olfactory ventricular neuroepithelium confirmed that Reelin-expressing cells migrate ventrally from this region and failed to see dorsally-migrating cells, thus coinciding with the results of Ceci et al. (2012). We propose that the discrepancy between the results of Bielle et al. (2005) and those of Ceci et al. (2012) and our own results, stem from differences between the culture methods employed. While the former performed the fluorescent tracing experiments in coronal slices of embryonic telencephalon, the present study and those of Ceci et al. (2012) were performed in whole embryos in culture. We believe that coronal slices hamper the antero-posterior migration and constrain the possibilities of migration to dorso-ventrally oriented routes. Because whole-embryo culture allows the migration of cells with no exogenous constrains, it represents a more natural experimental system than cultured slices.

Complementary evidence put forward by Bielle et al. (2005) for the existence of the dorsally-migrating CR population relies on *Dbx1* expression present in the VP/PSB and absent from other medial telencephalic regions. To corroborate this, we analyzed *Dbx1* mRNA expression, confirming it in the VP/PSB VZ and the septum, and revealed an additional domain of expression in the rostral region of the cortical hem. Since the cortical hem has been demonstrated by several groups to be the main source of CR cells (Takiguchi-Hayashi et al., 2004; García-Moreno et al., 2007; Ceci et al., 2010) and the studies of Ceci et al. (2012) failed to detect CR cell migration from the septum to the neocortex, we propose that the Dbx1^+^ CR cells detected by Bielle et al. (2005) could derive from the rostral-most aspect of the cortical hem and not from the septum and VP/PSB themselves.

Our results additionally resemble those obtained by Ceci et al. (2012) in that approximately 29% of the labeled cells originating in the ventral pallium expressed Reelin. However, this was not the case for the rest of the markers, as Ceci et al. (2012) identified 45% of cells expressing Tbr1 while 10% expressed Calbindin; in this study 27% of cells expreseed Reelin, 6% expressed Tbr1 and 3% expressed Calbindin. Such differences could result from technical discrepancies in both embryo stage and labeling sites.

Overall, our results reveal novel populations of Reelin-expressing cells that migrate from the rostrolateral telencephalon and confirm that migration from the VP/PSB region occurs in ventral and caudal directions only. It is possible, however, that the CFDA-labeled cells originating in the VP contribute to early-born CR cell subsets from the LOT that migrate to the neocortex at mid-gestation, as shown recently (de Frutos et al., 2016). Hence, these results add to the complex picture of the olfactory region of the telencephalon as a territory of extensive cell displacement during embryonic development.

## Author Contributions

AM and AV-E conceived the project and designed the experiments; MDF-P performed the experiments and analyzed the data; AM, MDF-P and AV-E wrote the article; AV-E supervised the project.

## Conflict of Interest Statement

The authors declare that the research was conducted in the absence of any commercial or financial relationships that could be construed as a potential conflict of interest.

## Footnotes

^1^https://imagej.net

## References

[B2] Bielle F. Griveau A. Narboux-Nême N. Vigneau S. Sigrist M. Arber S.. (2005). Multiple origins of Cajal-Retzius cells at the borders of the developing pallium. Nat. Neurosci. 8, 1002–1012. 10.1038/nn1511 16041369

[B3] Bulfone A. Smiga S. M. Shimamura K. Peterson A. Puelles L. Rubenstein J. L. (1995). *T-brain-1*: a homolog of *Brachyury* whose expression defines molecularly distinct domains within the cerebral cortex. Neuron 15, 63–78. 10.1016/0896-6273(95)90065-9 7619531

[B4] Carceller H. Rovira-Esteban L. Nacher J. Castrén E. Guirado R. (2016). Neurochemical phenotype of reelin immunoreactive cells in the piriform cortex layer II. Front. Cell Neurosci. 10:65. 10.3389/fncel.2016.00065 27013976 PMC4785191

[B5] Ceci M. L. López-Mascaraque L. de Carlos J. A. (2010). The influence of the environment on Cajal-Retzius cell migration. Cereb. Cortex 20, 2348–2360. 10.1093/cercor/bhp305 20100897

[B6] Ceci M. L. Pedraza M. de Carlos J. A. (2012). The embryonic septum and ventral pallium, new sources of olfactory cortex cells. PLoS One 7:e44716. 10.1371/journal.pone.0044716 22984546 PMC3439381

[B7] Chao D. L. Ma L. Shen K. (2009). Transient cell-cell interactions in neural circuit formation. Nat. Rev. Neurosci. 10, 262–271. 10.1038/nrn2594 19300445 PMC3083859

[B8] Chowdhury T. G. Jimenez J. C. Bomar J. M. Cruz-Martin A. Cantle J. P. Portera-Cailliau C. (2010). Fate of Cajal-Retzius neurons in the postnatal mouse neocortex. Front. Neuroanat. 4:10. 10.3389/neuro.05.010.2010 20339484 PMC2845061

[B9] D’Arcangelo G. Miao G. G. Chen S. C. Soares H. D. Morgan J. I. Curran T. (1995). A protein related to extracellular matrix proteins deleted in the mouse mutant reeler. Nature 374, 719–723. 10.1038/374719a0 7715726

[B10] de Carlos J. A. López-Mascaraque L. Valverde F. (1996). Dynamics of cell migration from the lateral ganglionic eminence in the rat. J. Neurosci. 16, 6146–6156. 8815897 10.1523/JNEUROSCI.16-19-06146.1996 PMC6579193

[B11] de Frutos C. A. Bouvier G. Arai Y. Thion M. S. Lokmane L. Keita M.. (2016). Reallocation of olfactory Cajal-Retzius cells shapes neocortex architecture. Neuron 92, 435–448. 10.1016/j.neuron.2016.09.020 27693257

[B12] Diodato A. Ruinart de Brimont M. Yim Y. S. Derian N. Perrin S. Pouch J.. (2016). Molecular signatures of neural connectivity in the olfactory cortex. Nat. Commun. 7:12238. 10.1038/ncomms12238 27426965 PMC4960301

[B13] García-Moreno F. López-Mascaraque L. De Carlos J. A. (2007). Origins and migratory routes of murine Cajal-Retzius cells. J. Comp. Neurol. 500, 419–432. 10.1002/cne.21128 17120279

[B14] García-Moreno F. López-Mascaraque L. de Carlos J. A. (2008). Early telencephalic migration topographically converging in the olfactory cortex. Cereb. Cortex 18, 1239–1252. 10.1093/cercor/bhm154 17878174

[B15] Grkovic I. Anderson C. R. (1997). Calbindin D28K-immunoreactivity identifies distinct subpopulations of sympathetic pre- and postganglionic neurons in the rat. J. Comp. Neurol. 386, 245–259. 10.1002/(sici)1096-9861(19970922)386:2<245::aid-cne6>3.0.co;2-1 9295150

[B16] Hevner R. F. Daza R. A. Rubenstein J. L. Stunnenberg H. Olavarria J. F. Englund C. (2003a). Beyond laminar fate: toward a molecular classification of cortical projection/pyramidal neurons. Dev. Neurosci. 25, 139–151. 10.1159/000072263 12966212

[B17] Hevner R. F. Neogi T. Englund C. Daza R. A. Fink A. (2003b). Cajal-Retzius cells in the mouse: transcription factors, neurotransmitters and birthdays suggest a pallial origin. Dev. Brain Res. 141, 39–53. 10.1016/s0165-3806(02)00641-7 12644247

[B18] Hevner R. F. Shi L. Justice N. Hsueh Y. Sheng M. Smiga S.. (2001). Tbr1 regulates differentiation of the preplate and layer 6. Neuron 29, 353–366. 10.1016/S0896-6273(01)00211-2 11239428

[B19] Huilgol D. Tole S. (2016). Cell migration in the developing rodent olfactory system. Cell. Mol. Life Sci. 73, 2467–2490. 10.1007/s00018-016-2172-7 26994098 PMC4894936

[B20] Huilgol D. Udin S. Shimogori T. Saha B. Roy A. Aizawa S.. (2013). Dual origins of the mammalian accessory olfactory bulb revealed by an evolutionarily conserved migratory stream. Nat. Neurosci. 16, 157–165. 10.1038/nn.3297 23292680

[B21] Marin O. Rubenstein J. L. (2001). A long, remarkable journey: tangential migration in the telencephalon. Nat. Rev. Neurosci. 2, 780–790. 10.1038/35097509 11715055

[B22] Meyer E. A. Illig K. R. Brunjes P. C. (2006). Differences in chemo- and cytoarchitectural features within pars principalis of the rat anterior olfactory nucleus suggest functional specialization. J. Comp. Neurol. 498, 786–795. 10.1002/cne.21077 16927267 PMC1592518

[B23] Meyer G. Cabrera Socorro A. Perez Garcia C. G. Martinez Millan L. Walker N. Caput D. (2004). Developmental roles of p73 in Cajal-Retzius cells and cortical patterning. J. Neurosci. 24, 9878–9887. 10.1523/JNEUROSCI.3060-04.2004 15525772 PMC6730229

[B24] Meyer G. Goffinet A. M. Fairen A. (1999). What is a Cajal-Retzius cell? a reassessment of a classical cell type based on recent observations in the developing neocortex. Cereb. Cortex 9, 765–775. 10.1093/cercor/9.8.765 10600995

[B25] Miquelájauregui A. Varela-Echavarría A. Ceci M. L. García-Moreno F. Ricaño I. Hoang K.. (2010). LIM-homeobox gene Lhx5 is required for normal development of Cajal-Retzius cells. J. Neurosci. 30, 10551–10562. 10.1523/JNEUROSCI.5563-09.2010 20685998 PMC2927820

[B26] Ogawa M. Miyata T. Nakajima K. Yagyu K. Seike M. Ikenaka K.. (1995). The reeler gene-associated antigen on Cajal-Retzius neurons is a crucial molecule for laminar organization of cortical neurons. Neuron 14, 899–912. 10.1016/0168-9525(95)90534-0 7748558

[B27] Pedraza M. de Carlos J. A. (2012). A further analysis of olfactory cortex development. Front. Neuroanat. 6:35. 10.3389/fnana.2012.00035 22969708 PMC3430874

[B28] Ruiz-Reig N. Andrós B. Huilgol D. Grove E. A. Tissir F. Tole S.. (2017). Lateral thalamic eminence: a novel origin for mGluR1/lot cells. Cereb. Cortex 27, 2841–2856. 10.1093/cercor/bhw126 27178193 PMC6248457

[B29] Sarma A. A. Richard M. B. Greer C. A. (2011). Developmental dynamics of piriform cortex. Cereb. Cortex 21, 1231–1245. 10.1093/cercor/bhq199 21041199 PMC3140179

[B30] Schwob J. E. Haberly L. B. Price J. L. (1984). The development of physiological responses of the piriform cortex in rats to stimulation of the lateral olfactory tract. J. Comp. Neurol. 223, 223–237. 10.1002/cne.902230206 6707249

[B31] Soriano E. Del Río J. A. (2005). The cells of cajal-retzius: still a mystery one century after. Neuron 46, 389–394. 10.1016/j.neuron.2005.04.019 15882637

[B32] Suzuki N. Bekkers J. M. (2006). Neural coding by two classes of principal cells in the mouse piriform cortex. J. Neurosci. 26, 11938–11947. 10.1523/JNEUROSCI.3473-06.2006 17108168 PMC6674875

[B33] Suzuki N. Bekkers J. M. (2011). Two layers of synaptic processing by principal neurons in piriform cortex. J. Neurosci. 31, 2156–2166. 10.1523/JNEUROSCI.5430-10.2011 21307252 PMC6633060

[B34] Takiguchi-Hayashi K. Sekiguchi M. Ashigaki S. Takamatsu M. Hasegawa H. Suzuki-Migishima R.. (2004). Generation of reelin-positive marginal zone cells from the caudomedial wall of telencephalic vesicles. J. Neurosci. 24, 2286–2295. 10.1523/JNEUROSCI.4671-03.2004 14999079 PMC6730420

[B35] Tissir F. Ravni A. Achouri Y. Riethmacher D. Meyer G. Goffinet A. M. (2009). DeltaNp73 regulates neuronal survival *in vivo*. Proc. Natl. Acad. Sci. U S A 106, 16871–16876. 10.1073/pnas.0903191106 19805388 PMC2757832

[B36] Varela-Echavarría A. Pfaff S. L. Guthrie S. (1996). Differential expression of LIM homeobox genes among motor neuron subpopulations in the developing chick brain stem. Mol. Cell. Neurosci. 8, 242–257. 10.1006/mcne.1996.0061 9000439

[B37] Yamazaki H. Sekiguchi M. Takamatsu M. Tanabe Y. Nakanishi S. (2004). Distinct ontogenic and regional expressions of newly identified Cajal-Retzius cell-specific genes during neocorticogenesis. Proc. Natl. Acad. Sci. U S A 101, 14509–14514. 10.1073/pnas.0406295101 15452350 PMC521974

[B38] Zhao Y. Sheng H. Z. Amini R. Grinberg A. Lee E. Huang S.. (1999). Control of hippocampal morphogenesis and neuronal differentiation by the LIM homeobox gene *Lhx5*. Science 284, 1155–1158. 10.1126/science.284.5417.1155 10325223

[B39] Zimmer C. Lee J. Griveau A. Arber S. Pierani A. Garel S.. (2010). Role of Fgf8 signalling in the specification of rostral Cajal-Retzius cells. Development 137, 293–302. 10.1242/dev.041178 20040495 PMC2799162

